# ATR inhibitor AZD6738 enhances the antitumor activity of radiotherapy and immune checkpoint inhibitors by potentiating the tumor immune microenvironment in hepatocellular carcinoma

**DOI:** 10.1136/jitc-2019-000340

**Published:** 2020-05-26

**Authors:** Hailong Sheng, Yan Huang, Yazhi Xiao, Zhenru Zhu, Mengying Shen, Peitao Zhou, Zeqin Guo, Jian Wang, Hui Wang, Wencong Dai, Wanjun Zhang, Jingyuan Sun, Chuanhui Cao

**Affiliations:** 1 Department of Radiation Oncology, Southern Medical University Nanfang Hospital, Guangzhou, Guangdong, China; 2 Hepatology Unit and Department of Infectious Diseases, Southern Medical University Nanfang Hospital, Guangzhou, Guangdong, China; 3 Department of Stomatology, No 2 Hospital of Baoding, Baoding, Hebei, China

**Keywords:** immunology, oncology, tumors, radiotherapy

## Abstract

**Background:**

Radioimmunotherapy has a promising antitumor effect in hepatocellular carcinoma (HCC), depending on the regulatory effect of radiotherapy on tumor immune microenvironment. Ionizing radiation (IR)-induced DNA damage repair (DDR) pathway activation leads to the inhibition of immune microenvironment, thus impairing the antitumor effect of radioimmunotherapy. However, it is unclear whether inhibition of the DDR pathway can enhance the effect of radioimmunotherapy. In this study, we aim to explore the role of DDR inhibitor AZD6738 on the combination of radiotherapy and immune checkpoint inhibitors (ICIs) in HCC.

**Methods:**

C57BL/6 mouse subcutaneous tumor model was used to evaluate the ability of different treatment regimens in tumor growth control and tumor recurrence inhibition. Effects of each treatment regimen on the alterations of immunophenotypes including the quantification, activation, proliferating ability, exhaustion marker expression, and memory status were assessed by flow cytometry.

**Results:**

AZD6738 further increased radiotherapy-stimulated CD8^+^ T cell infiltration and activation and reverted the immunosuppressive effect of radiation on the number of Tregs in mice xenografts. Moreover, compared with radioimmunotherapy (radiotherapy plus anti-PD-L1 (Programmed death ligand 1)), the addition of AZD6738 boosted the infiltration, increased cell proliferation, enhanced interferon (IFN)-γ production ability of TIL (tumor-infiltrating lymphocyte) CD8^+^ T cells, and caused a decreasing trend in the number of TIL Tregs and exhausted T cells in mice xenografts. Thus, the tumor immune microenvironment was significantly improved. Meanwhile, triple therapy (AZD6738 plus radiotherapy plus anti-PD-L1) also induced a better immunophenotype than radioimmunotherapy in mice spleens. As a consequence, triple therapy displayed greater benefit in antitumor efficacy and mice survival than radioimmunotherapy. Mechanism study revealed that the synergistic antitumor effect of AZD6738 with radioimmunotherapy relied on the activation of cyclic GMP–AMP synthase /stimulator of interferon genes (cGAS/STING) signaling pathway. Furthermore, triple therapy led to stronger immunologic memory and lasting antitumor immunity than radioimmunotherapy, thus preventing tumor recurrence in mouse models.

**Conclusions:**

Our findings indicate that AZD6738 might be a potential synergistic treatment for radioimmunotherapy to control the proliferation of HCC cells, prolong survival, and prevent tumor recurrence in patients with HCC by improving the immune microenvironment.

## Background

Hepatocellular carcinoma (HCC) is the fifth most common cancer and the second leading cause of cancer-related death worldwide.^1^ The prognosis of patients with HCC is poor because most patients are diagnosed at the advanced stage when surgery and treatment are ineffective. Local therapy for advanced HCC is currently recommended by the National Comprehensive Cancer Network guidelines. Radiotherapy, as an indispensable local treatment method for cancers, plays an increasingly important role in the treatment of inoperable HCC.^2–4^ Radiotherapy combined with systematic treatment has been proven to be an effective strategy in many types of cancers (eg, lung and rectal cancers).^5^ However, the combination of radiotherapy with systematic treatment such as sorafenib, which is a multitarget tyrosine kinase inhibitor and recommended as the first-line treatment for patients with advanced HCC, did not dramatically improve the survival of patients with HCC.^6^ Given the poor prognosis of patients with HCC and current limited adjuvant systematic treatments for radiotherapy, novel therapeutic strategies are urgently needed to be explored.

In recent years, immune checkpoint inhibitors (ICIs) have been demonstrated to have a positive therapeutic effect on patients with advanced HCC, by yielding about 20% disease control rate.^7 8^ There are increasing substantial preclinical studies showing that radiotherapy combined with ICIs improve the control rate and reduce the recurrence rate of multiple types of cancers, including HCC.^9^ Therefore, it appears that the combination of radiotherapy and ICIs may become a promising treatment intervention for patients with advanced HCC in the future. It is well documented that radiation enhances the curative effect of ICIs by modulating the cancer immune microenvironment.^10^ For example, radiation induces DNA damage in cancer cells and facilitates dsDNA (double-strand DNA) release, thus activating the cGAS/STING signaling pathway; as a consequence, innate immunity is stimulated and lymphocyte infiltration is increased in cancer tissues.^11^ Meanwhile, by destroying cancer cells and promoting the antigen presentation, radiation activates immunity to induce lymphocyte infiltration and improve the efficacy of immunotherapy.^12–14^

However, other than simulative effect, radiation also has suppressive effect on the tumor environment. DNA damage repair (DDR) pathway activation triggered by radiation-induced DNA damage upregulates the expression of CTLA-4 and PD-L1, which creates an immunosuppressive microenvironment in tumor tissues.^15^ Ataxia telangiectasia and Rad3-related protein (ATR) is an essential kinase in the DDR transduction signaling pathway. AZD6738, a specific ATR inhibitor, has been demonstrated to potentiate radiation-induced lymphocyte infiltration and lymphocyte activation in tumor tissues, thus achieving a more potent anticancer effect.^16 17^ Moreover, AZD6738 is currently under investigations in 10 clinical trials in combination with either chemotherapy or radiotherapy. In the light of the previously described information, it is becoming increasingly important to explore whether AZD6738 could increase the therapeutic effect of radiotherapy combined with ICIs therapy. Therefore, the aim of this study is to comprehensively characterize the role of the AZD6738 on the modulation of tumor immune microenvironment and the treatment efficacy of radioimmunotherapy. Our data suggested a novel possible therapeutic approach for patients with advanced HCC, and this treatment paradigm merits evaluation in clinical trials in the future.

## Methods

### Cell culture

Hepa 1–6 cells (a C57/L murine liver cancer cell line) and H22 cells (a BALB/c murine liver cancer cell line) were obtained from the Shanghai Institute of Biochemistry and Cell Biology. Hepa 1–6 cells were cultured in DMEM (dulbecco's modified eagle medium) with 10% FBS (fetal bovine serum). H22 cells were cultured in RPMI-1640 (Roswell Park Memorial Institute-1640) medium with 10% FBS.

### Establishment of subcutaneous tumor models

Female C57BL/6 mice (4–6 weeks old) or BALB/c mice (4–6 weeks old) were provided by the Southern Medical University Animal Center. Hepa 1–6 cells (1×10^7^) were implanted subcutaneously in the right hind flank of the C57BL/6 mice. H22 cells (5×10^6^) were implanted subcutaneously in the right hind flank of the BALB/c mice. Tumor volumes were calculated using a standard formula: length×width^2^/2. Radiation was administered when tumors reached approximately 200 mm^3^. Tumor volume was measured every 5 days. For ethical considerations, mice were sacrificed when the tumor volumes reached 1000 mm^3^. Endpoint day was designated as the 60th day after commencement of treatment.

### Establishment of orthotopic liver cancer models

C57BL/6 mice (4–6 weeks old) were anesthetized by intraperitoneal injection with 2% sodium pentobarbital solution. After iodophor disinfection, liver was exposed, and 1×10^6^ Hepa 1–6 cells were slowly injected into the liver, followed by closure of the abdominal cavity. MRI was performed to visualize progression of the orthotopic HCC tumor, with treatment administered when the maximum diameter of the tumor reached 3–5 mm. After 40 days of treatment, the mice were sacrificed, and the livers were removed.

### Mice treatments

Mice received three fractions of 6 Gy on days 1, 3, and 5 from the start of treatment. Imaging and radiation were performed using a small animal radiation research platform (SARRP, Xstrahl). Mice were intragastrically administered AZD6738 (S7693, Selleck; 75 mg/kg) on days 1, 3, and 5 from the start of treatment. For the PD-L1 blockade experiment, 200 µg anti–PD-L1 (Clone:10F.9G2, Catalog #: BE0101) was administered intraperitoneally on days 1, 4, and 7 from the start of treatment. Mice were intraperitoneally injected with C-176 (S6575, Selleck; 5 mg/kg/day) daily from 1 week before radiation until the complete of radiation.

### Blood analysis

Peripheral blood samples were collected from the retro-orbital sinus on days 1, 4, and 7 after treatment initiation. Alanine aminotransferase (ALT) and serum creatinine (Scr) were detected using an animal biochemical analyzer.

### Statistical analysis

Statistical comparisons between three or more groups were performed using one-way analysis of variance (ANOVA), followed by Tukey’s multiple comparison test. For mouse subcutaneous tumor volume experiments, statistical analysis was performed using mixed-effects model, followed by Tukey’s multiple comparison test. Survival curves for different groups of mice were generated using the Kaplan-Meier method. Survival data were compared using the log-rank Mantel-Cox test. P<0.05 was considered statistically significant. All graphs were plotted using GraphPad Prism V.7.

## Results

### AZD6738 increases CD8^+^ T cell infiltration and activation following radiation

A previous report showed that inhibition of the DDR pathway reverts radiation-induced PD-L1 upregulation, suggesting that a DDR inhibitor might attenuate a radiation-induced immune microenvironment. We first tested the effect of AZD6738 on the expression level of PD-L1 in HCC cells treated with AZD6738, radiation, or the combination. We found that at 24 and 48 hours, radiation alone significantly upregulated PD-L1 expression (fold-change in PD-L1 median fluorescence intensity±SD, radiation vs vehicle: 24 hours, 2.02±0.15-fold, p<0.0001; 48 hours, 1.58±0.20-fold, p<0.0001). And the addition of AZD6738 to radiation decreased the radiation-induced PD-L1 upregulation (radiation vs AZD6738 plus radiation: 24 hours, 2.02±0.15-fold vs 1.05±0.02-fold, p<0.0001; 48 hours, 1.58±0.20-fold vs 0.92±0.07-fold, p<0.0001). At 72 hours, no difference was observed between radiation and AZD6738 with radiation (online supplementary figure 2).

10.1136/jitc-2019-000340.supp1 Supplementary data

**Figure 1:**
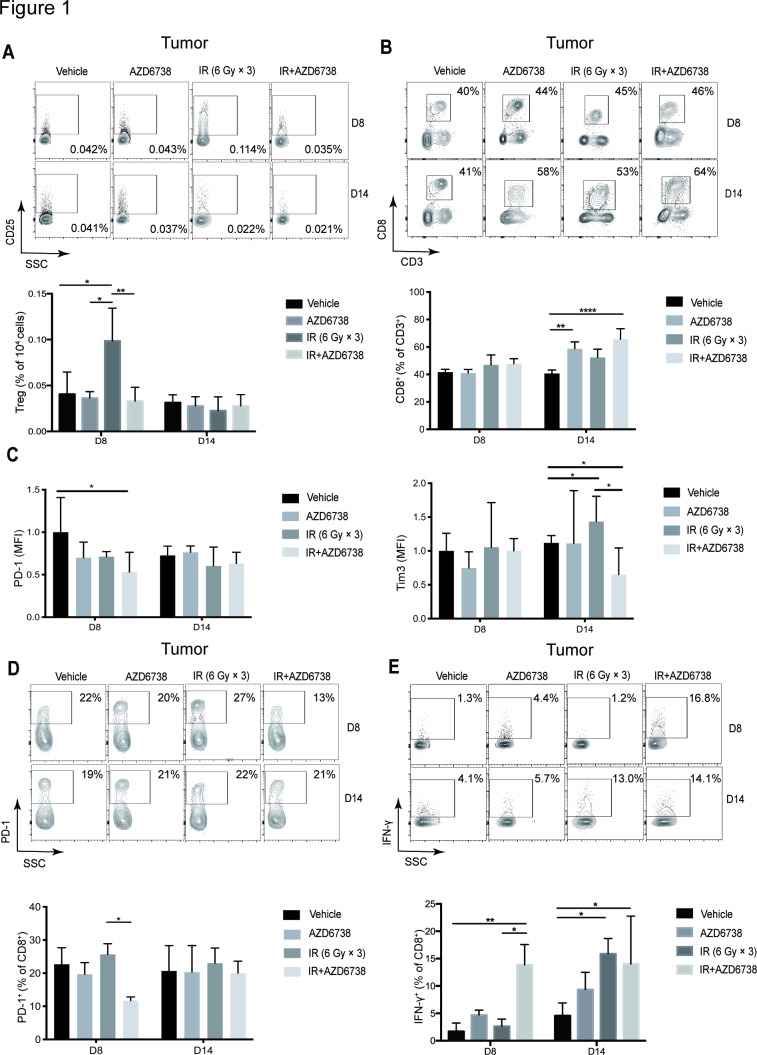
AZD6738 improves immune microenvironment in mice hepatocellular carcinoma (HCC) tissues after radiotherapy. (A) Representative contour plots depicting the percentages of tumor-infiltrating (TIL) Tregs (CD25^+^Foxp3^+^), and the quantitation of the number of TIL Tregs per 10^4^ cells stained at days 8 and 14 was shown. (B) Representative contour plots and quantification of CD8^+^/CD3^+^ ratios at days 8 and 14. (C) Quantitation of PD-1 and Tim3 median fluorescence intensity in TIL CD8^+^ T cells at days 8 and 14. (D) Representative contour plots and quantification of the percentage of TIL PD-1^+^ CD8^+^ T cells at days 8 and 14. (E) Representative contour plots and quantification of TIL CD8^+^ T cells that elicit IFN-γ following stimulation with PMA/ionomycin at days 8 and 14. Data represent the mean±SD for (A)-(E), statistical analysis was performed by using one-way analysis of variance with Tukey’s multiple comparisons test. *p<0.05; **p<0.005;+ ****p<0.0001. IFN-γ, interferon-γ; IR, ionizing radiation; PMA, phorbol 12-myristate 13-acetate.

Next, we explored the role of AZD6738 on immune microenvironment in vivo. Hepa 1–6 cells were implanted on the flanks of C57BL/6 mice. When the subcutaneous tumors grew to 200 mm^3^, we treated the mice with AZD6738, radiation, or the combination. At day 8, radiation alone increased the percentage of tumor-infiltrating lymphocyte (TIL) Tregs (0.099%±0.036% radiation vs 0.041%±0.024% vehicle, p=0.0188). However, AZD6738 plus radiation reduced the percentage of Tregs compared with radiation alone (0.033%±0.015% AZD6738 plus radiation vs radiation, p=0.0072). No differences were observed on day 14 (figure 1A). Moreover, addition of AZD6738 to radiation significantly increased the proportion of CD8^+^ T cells compared with vehicle on day 14 (65.7%±7.6% AZD6738 plus radiation vs 40.7%± 2.5% vehicle, p＜0.0001; figure 1B). At days 8 and 14, no differences in the proportion of CD8^+^ T cells were observed between vehicle and radiation.

Next, we determined the activation status of tumor-infiltrating CD8^+^ T cells. We investigated expression levels of PD-1 and Tim3 in CD8^+^ T cells. At day 8, we found that administration of AZD6738 plus radiation significantly downregulated PD-1 expression (fold-change in median fluorescence intensity±SD, AZD6738 plus radiation vs vehicle: 0.53±0.23-fold; p=0.0381) but showed no effect on Tim3 expression. At day 14, radiation treatment upregulated Tim3 expression (fold-change in median fluorescence intensity±SD, radiation vs vehicle: 1.43±0.37-fold; p=0.0112), and administration of AZD6738 plus radiation significantly downregulated Tim3 expression (AZD6738 plus radiation: 0.65±0.39-fold vs radiation; p=0.0413), whereas no differences were observed in PD-1 expression (figure 1C). At day 8, AZD6738 with radiation reduced the percentage of TIL CD8^+^PD-1^+^ T cells in comparison with radiation alone (11.7%±1.2% AZD6738 plus radiation vs 25.7%±3.2% radiation alone, p=0.0227). No differences were observed at day 14 (figure 1D).

At day 8, AZD6738 plus radiotherapy increased the percentage of TIL CD8^+^ T cells that produced IFN-γ compared with radiation alone (13.9%±3.7% AZD6738 with radiation vs 2.6%±1.3% radiation, p=0.0151). At day 14, compared with vehicle, both radiation alone and AZD6738 plus radiation increased the percentage of TIL CD8^+^ T cells that produced IFN-γ (figure 1E). In summary, AZD6738 potentiated the effect of radiation on reducing the number of exhausted T cells and increasing the cell-killing activity of CD8^+^ T cells, meanwhile, reverted radiation-induced Tregs infiltration.

### AZD6738 increase the antitumor efficacy of radiation combined with anti-PD-L1 and prolongs tumor-bearing mice survival

**Figure 2:**
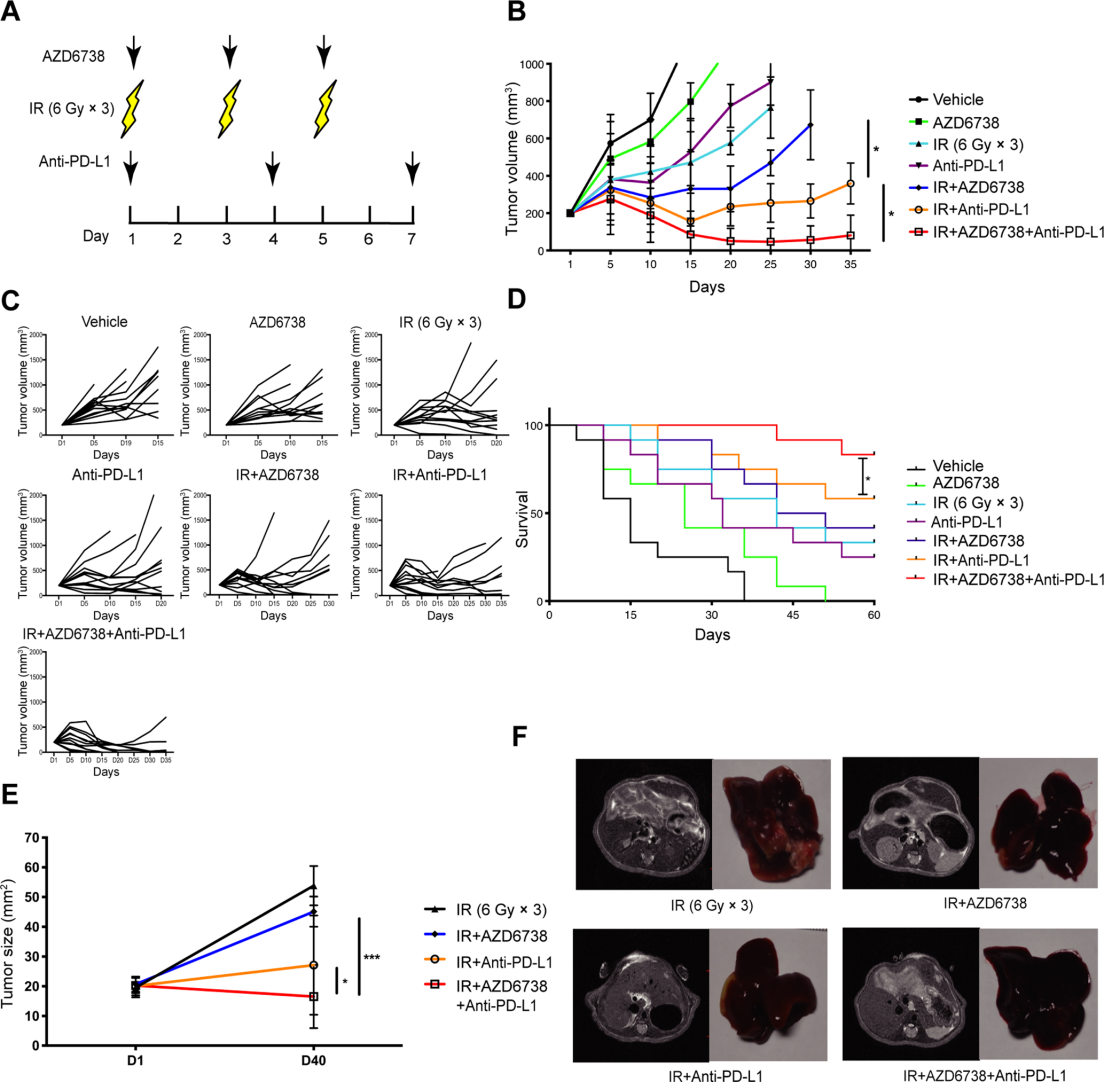
Addition of AZD6738 potentiates radiation combined with anti-PD-L1 therapy in mice tumor growth control and survival improvement. (A) Schematic showing schedules of ionizing radiation (IR), AZD6738, and anti-PD-L1 treatment. (B–C) Response of the subcutaneous tumors (B) or individual tumor volumes (C) to the indicated treatment regimens. n=12 in each group. Data represent the mean±SEM. (D) Survival plots for each treatment regimens were shown. (E) Response of the orthotopic hepatocellular carcinoma (HCC) tumors to treatment with the indicated treatment regimens. n=5 in each group. Data represent the mean±SEM. (F) Representative images of mice liver MRI before treatment and the corresponded isolated liver samples after treatment. For (B), statistical analyses were performed using a mixed-effects model, followed by Tukey’s multiple comparison test. For (D), survival data were compared using the log-rank Mantel-Cox test. For (E), statistical analyses were performed using one-way analysis of variance with Tukey’s multiple comparisons test. *p<0.05; ***p<0.0005.

Because AZD6738 improved the radiation-induced immunosuppressive microenvironment, it is possible that its use could also enhance the antitumor effect of radiotherapy combined with ICIs. To determine the treatment efficacy of AZD6738, the Hepa 1–6 and H22 tumor-bearing mice were treated with varied treatment regimens (figure 2A). In Hepa 1–6 tumor-bearing mice models, for vehicle and AZD6738 alone group, the designated tumor volume endpoint (1000 mm^3^) was reached at days 15 and 20, respectively. Both radiation alone and anti-PD-L1 alone groups reached the endpoint on day 25. AZD6738 plus radiation group reached the endpoint at day 30. Until day 35, anti-PD-L1 plus radiation (radioimmunotherapy) group and radiotherapy combined with anti-PD-L1 and AZD6738 (triple therapy) group still did not reach the endpoint yet. Furthermore, compared with radioimmunotherapy, triple therapy significantly inhibited tumor growth (mean tumor volume±SEM: 81±59 mm^3^ triple therapy vs 369±147 mm^3^ radioimmunotherapy, p=0.0172; figure 2B, C). Similarly, in H22-tumor-bearing mouse models, triple therapy significantly inhibited tumor growth relative to radioimmunotherapy (mean tumor volume±SEM: 438±61 mm^3^ triple therapy vs 771±72 mm^3^ radioimmunotherapy; p=0.0301) (online supplementary figure 3). In Hepa 1–6 tumor-bearing mice models, the average survival time of vehicle group was 15 days, and these of the AZD6738 alone, anti-PD-L1 alone, and radiation alone groups were 25, 32, and 42 days. Compared with radiation alone, the addition of AZD6738 or anti-PD-L1 to radiation did not further increase the mice survival. Meanwhile, triple therapy significantly prolonged survival compared with radiation alone or radioimmunotherapy. In addition, triple therapy showed higher complete response rate than radioimmunotherapy (83% triple therapy vs 58% radioimmunotherapy; figure 2D). Therefore, the addition of AZD6738 to radioimmunotherapy significantly inhibited tumor growth, increased complete response rate, and prolonged the survival time of mice.

Importantly, to better replicate a clinical scenario, we treated orthotopic HCC-bearing mice with various regimens, subsequently observing that triple therapy exhibited better antitumor efficacy than radioimmunotherapy (mean tumor volume±SEM: 16.7±9.4 mm^3^ triple therapy vs 32.9±17.0 mm^3^ radioimmunotherapy, p=0.0421; figure 2E, F).

More importantly, the addition of AZD6738 was well tolerated without abnormal levels of ALT and Scr in mice blood samples. These data suggested that AZD6738 increased the antitumor effect of radioimmunotherapy without impairing liver and kidney functions in mice (online supplementary figure 4).

### AZD6738 impacts T cell infiltration following radioimmunotherapy

**Figure 3:**
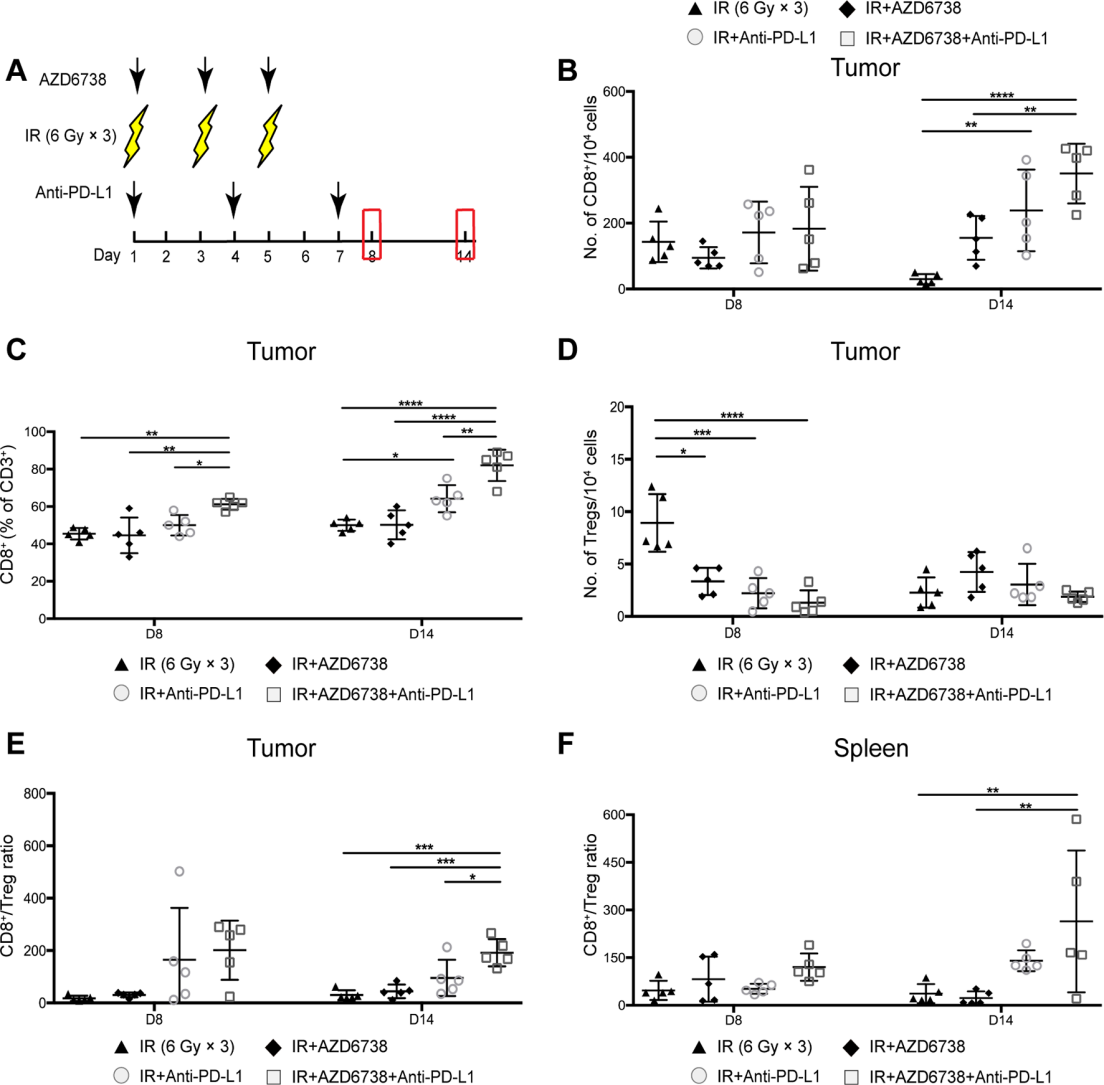
Triple therapy impacts T cell infiltration in mice tumors and spleen. (A) Schematic shows schedules of triple therapy including ionizing radiation (IR), AZD6738, and anti-PD-L1. The red frames indicated the time points for lymphocyte analyses in mice tumors or spleens. (B) Quantitation of the number of tumor-infiltrating lymphocyte (TIL) CD8^+^ T cells per 10^4^ cells at days 8 and 14. (C) Quantitation of CD8^+^/CD3^+^ ratios in mice tumors. (D) Quantitation of the number of TIL Tregs per 10^4^ cells. (E) Quantitation of CD8^+^/Treg ratios in mice tumors. (F) Quantitation of CD8^+^/Treg ratios in mice spleens. Data represent the mean±SD. For (B)–(F), statistical analysis was performed by using one-way analysis of variance with Tukey’s multiple comparisons test. *p<0.05; **p<0.005; ***p<0.0005; ****p<0.0001.

As the antitumor effect of the triple therapy is better than that of radioimmunotherapy, we need to identify the effect of AZD6738 on the alterations in tumor immune microenvironment. Lymphocytes from the tumors and spleen were collected at days 8 and 14 (figure 3A). At day 14, both radioimmunotherapy and triple therapy significantly increased the number of TIL CD8^+^ T cells compared with radiation alone (30.2±15.2 radiation alone vs 238.8±124.3 radioimmunotherapy, p=0.0060; vs 350.6±90.8 triple therapy, p<0.0001; figure 3B). AZD6738 increased the numbers of TIL CD8^+^ T cells in triple therapy compared with that of in radioimmunotherapy, but the difference did not reach statistical significance. At day 14, anti-PD-L1 with radiation significantly increased the ratio of CD8^+^/CD3^+^ T cells compared with radiation alone (50.0%±3.0% radiation alone vs 64.2%±7.3% anti-PD-L1 with radiation, p=0.0239; figure 3C). Triple therapy further increased the ratio of CD8^+^/CD3^+^ T cells compared with anti-PD-L1 with radiation at days 8 and 14 (day 8, 61.2%±3.0% triple therapy vs 50.0%±5.5% radioimmunotherapy, p=0.0384; day 14, 82.0%±8.4% triple therapy vs 64.2%±7.3% radioimmunotherapy, p=0.0046; figure 3C).

Meanwhile, we examined the effect of AZD6738 on the number of TIL Tregs. On day 8, AZD6738 plus radiation, radioimmunotherapy, and triple therapy significantly inhibited the radiation-induced TIL Tregs infiltration (4.4±2.1 AZD6738 plus radiation vs 8.9±2.7 radiation, p=0.0111; 2.2±1.4 radioimmunotherapy vs radiation, p=0.0003; 1.1±1.3 triple therapy vs radiation, p<0.0001; figure 3D). At days 8 and 14, compared with AZD6738 plus radiation and anti-PD-L1 plus radiation, triple therapy further reduced the number of TIL Tregs, but the difference did not reach statistical significance (figure 3D). Although no difference in the number of TIL Tregs was observed between triple therapy and radioimmunotherapy, triple therapy significantly increased the CD8^+^/Treg ratio at day 14 compared with radioimmunotherapy (191.5±51.8 triple therapy vs 95.7±69.6 radioimmunotherapy, p=0.0222; figure 3E). From day 8 to day 14, the CD8^+^/Treg ratio in the spleen increased in triple therapy. No differences in CD8^+^/Treg ratio among different groups were observed on day 8. At day 14, only triple therapy significantly upregulated CD8^+^/Treg ratio than that of radiation alone group in spleen (264.1±223.2 triple therapy vs 10.2±5.8 radiation, p=0.0286; figure 3F).

### Triple therapy influences the proliferation of T cells in tumors and spleens of tumor-bearing mice

**Figure 4:**
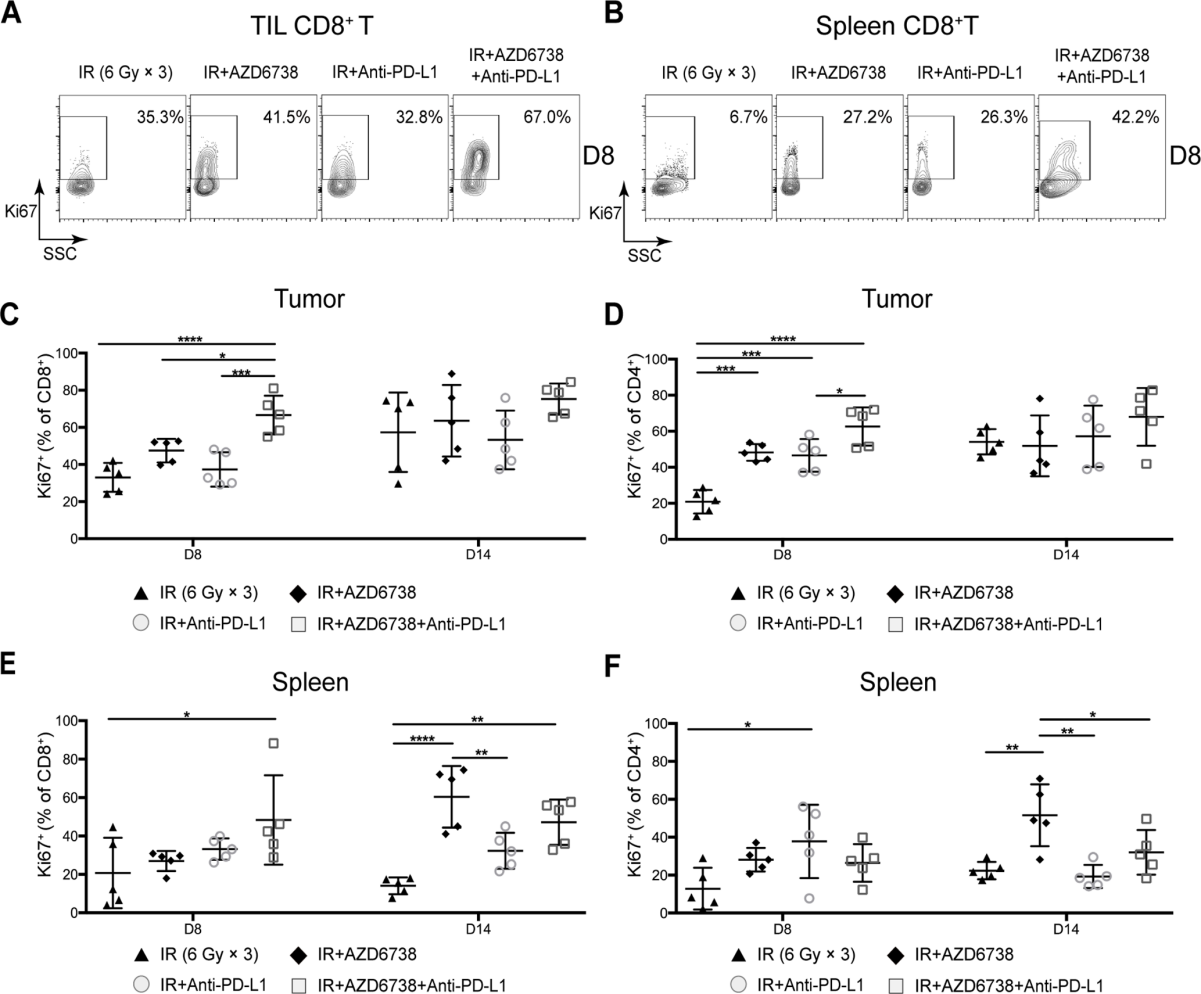
Triple therapy promotes the proliferation of splenic and tumor-infiltrating T cells. (A–B) Representative contour plots of Ki67^+^ expression on tumor-infiltrating lymphocyte (TIL) and splenic CD8^+^ T cells at day 8. (C–D) Quantitation of the percentages of proliferating (Ki67^+^) TIL CD8^+^ T cells (C) and TIL CD4^+^ T cells (D) at days 8 and 14. (E–F) Quantitation of the percentages of proliferating (Ki67^+^) splenic CD8^+^ T cells (E) and splenic CD4^+^ T cells (F) at days 8 and 14. Data represent the mean±SD. For (C)–(F), statistical analysis was performed by using one-way analysis of variance with Tukey’s multiple comparisons test. *p<0.05; **p<0.005; ***p<0.0005; ****p<0.0001. IR, ionizing radiation.

Next, we examined the proliferating (Ki67^+^) splenic and TIL T cell population from varied treatments. At day 8, compared with radiation alone, only triple therapy significantly increased the percentage of TIL CD8^+^ Ki67^+^ T cells (66.6%±10.5% triple therapy vs 47.5%±6.3% radiation p<0.0001; vs 47.5%±6.3% AZD6738 plus radiation, p=0.0136; vs 37.3%±9.3% radioimmunotherapy, p=0.0003; figure 4A). At day 14, the addition of AZD6738 to radioimmunotherapy did not impact the percentage of TIL CD8^+^ Ki67^+^T cells compared with that of radioimmunotherapy group (figure 4C). Similar phenomena were seen in spleens. Only triple therapy significantly increased the percentage of splenic CD8^+^ Ki67^+^ T cell at day 8 compared with radiation alone (60.0%±22.2% triple therapy vs 20.7%±18.3% vehicle, p=0.0253). No differences were observed at day 14 (figure 4B and E).

With respect to the percentage of TIL effector (Eff) T cells, at day 8, radioimmunotherapy and triple therapy significantly increased the percentage of TIL CD4^+^Ki67^+^ Eff T cells compared with radiation alone (20.9%±6.5% radiation vs 46.6%±9.1% radioimmunotherapy, p=0.0006; vs 42.7%±10.9% triple therapy, p<0.0001; figure 4D). Triple therapy further increased this percentage compared with that of radioimmunotherapy group (46.6%±9.1% radioimmunotherapy vs 42.7%±10.9% triple therapy, p=0.0282; figure 4D). No differences were observed between radioimmunotherapy and triple therapy at day 14 (figure 4D). Furthermore, at day 8, no differences in the percentage of splenic Ki67^+^CD4^+^ Eff T cells were observed between radioimmunotherapy and triple therapy (figure 4F). At day 14, the percentage of CD4^+^Ki67^+^ Eff T cells in triple therapy group increased compared with that of radioimmunotherapy, but the difference did not reach statistical significance (figure 4F).

### Triple therapy attenuates the expression of immune exhaustion markers in CD8^+^ T cells

**Figure 5:**
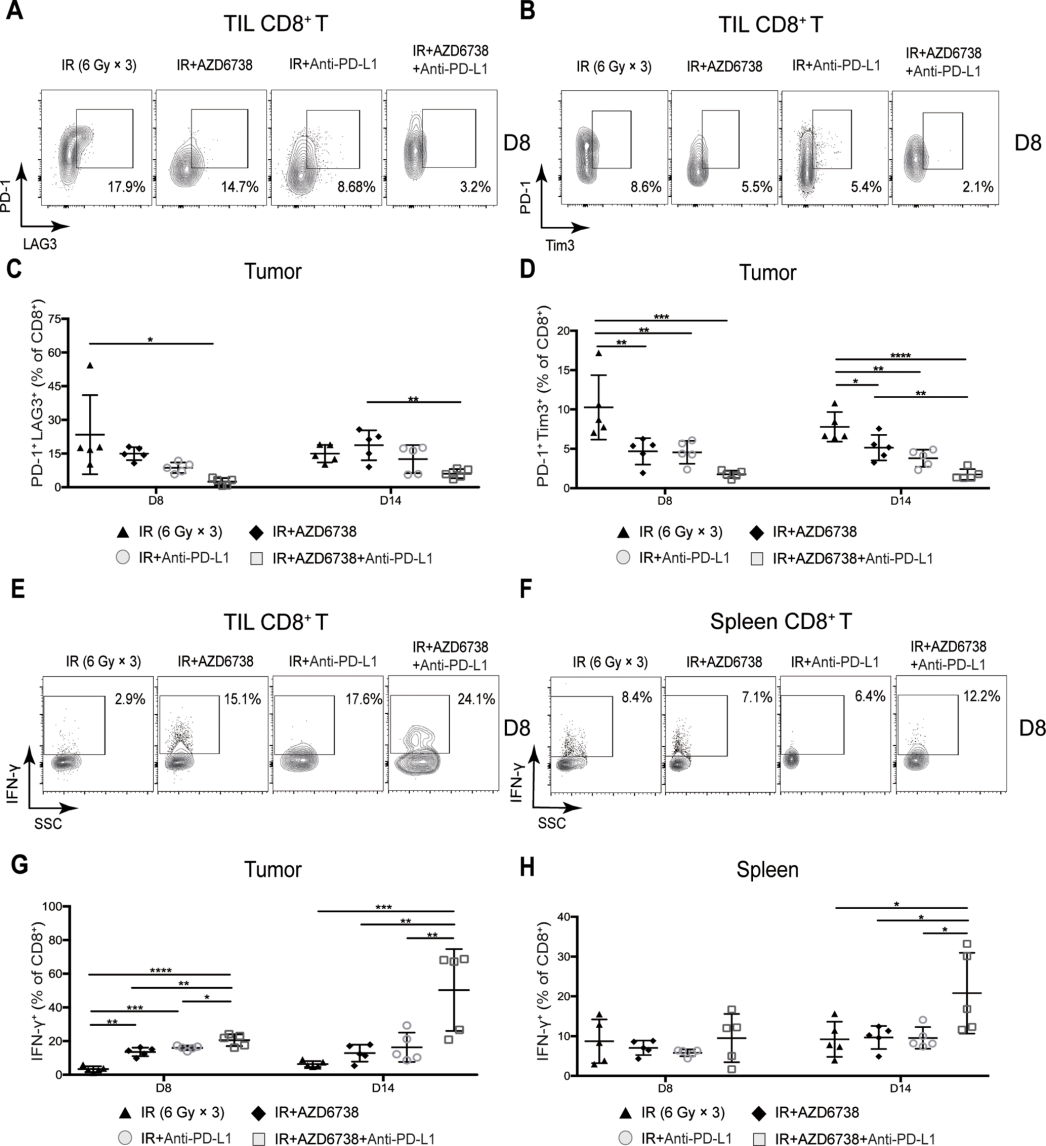
Triple therapy attenuates coexpression of CD8^+^ T cell exhaustion markers and promotes CD8^+^ T cell effector function in mice tumors. (A–B) Representative contour plots depicting PD-1 and LAG-3 coexpression and PD-1 and Tim-3 coexpression on tumor-infiltrating lymphocyte (TIL) CD8^+^ T cells at day 8. (C–D) Quantitation of the percentage of TIL CD8^+^ T cells coexpressing PD-1 and LAG-3 (C), and PD-1 and Tim-3 (D) at days 8 and 14. (E–F) Representative contour plots depicting IFN-γ expressing CD8^+^ T cells in mice spleens and tumors following stimulation with PMA/ionomycin at day 8. (G–H) Quantitation of the percentage of TIL (G) and splenic (H) CD8^+^ T cells that expressed IFN-γ at days 8 and 14. Data represent the mean±SD. For (C), (D), (G) and (H), statistical analysis was performed by using one-way analysis of variance with Tukey’s multiple comparisons test. *p<0.05; **p<0.005; ***p<0.0005; ****p<0.0001. IFN-γ, interferon-γ; IR, ionizing radiation.

Coexpression of T cell immune exhaustion markers, including PD-1, Tim-3, and LAG-3, can inhibit T cell antitumor function.^18–21^ Therefore, we evaluated the expression of T cell surface immune exhaustion markers in all treatment regimens. At day 8, compared with radiation alone, radioimmunotherapy did not impact the percentage of TIL CD8^+^PD-1^+^LAG3^+^ T cells, whereas triple therapy significantly reduced the percentage of these immunosuppressive T cells (2.5%±1.6% triple therapy vs 23.4%±17.6% radiation, p=0.0122; figure 5A and C). At day 14, the percentage of TIL CD8^+^PD-1^+^LAG3^+^ T cells was lower in triple therapy than that in AZD6738 plus radiation group or radioimmunotherapy group, although statistical differences were not achieved (figure 5C).

At day 8, we observed no differences in PD-1 or Tim3 expression. At day 14, we found that triple therapy significantly downregulated Tim3 expression (fold-change in median fluorescence intensity±SD, triple therapy vs radiation: 0.43±0.18-fold; p=0.103), whereas no differences were observed in PD-1 expression (online supplementary figure 5A, B. Triple therapy maintained a lower percentage of TIL CD8^+^PD-1^+^Tim3^+^T cells compared with AZD6738 plus radiation and radioimmunotherapy, but the difference did not reach statistical significance (figure 5B, D). There were no differences in the percentage of CD8^+^PD-1^+^LAG3^+^ and CD8^+^PD-1^+^Tim3^+^ T cells between triple therapy and radioimmunotherapy in the spleens (online supplementary figure 5C and D). In summary, compared with radioimmunotherapy, triple therapy can further reduce the coexpression of CD8^+^ T cell exhaustion markers in mice xenograft tumors and spleens.

### Triple therapy promotes CD8^+^ T cell effector function in mice tumors and spleens

To confirm whether the addition of AZD6738 to radioimmunotherapy can enhance effector function after reducing coexpression exhaustion markers of CD8^+^ T cells, we used phorbol 12-myristate 13-acetate (PMA) and ionomycin to stimulate IFN-γ production of CD8^+^ T cells. At day 8, adding either AZD6738 or anti-PD-L1 to radiation significantly increased the percentage of TIL CD8^+^ T cells that produced IFN-γ, and triple therapy further increases this percentage (20.5%±3.5% triple therapy vs 13.6%±2.6% AZD6738 plus radiation, p=0.0024; vs 15.6%±2.1% radioimmunotherapy, p=0.0317; figure 5E, G). At day 14, the percentage of TIL CD8^+^ T cells that produced IFN-γ remained the same as that of day 8 in mice treated with radiation alone, AZD6738 with radiation, and radioimmunotherapy; however, that of triple therapy was further increased, and it was significantly higher than that of the other treatment groups (50.3%±24.3% triple therapy vs 6.4%±1.8% radiation, p=0.0004; vs 12.8%±5.0% AZD6738 with radiation, p=0.0019 vs 16.3%±8.7% radioimmunotherapy, p=0.0044; figure 5G).

With respect to the percentage of CD8^+^ T cells that produced IFN-γ in mice spleens, no differences were observed among all treatments at day 8 (figure 5F, H). At day 14, the percentage in triple therapy was significantly higher than that in the other treatment groups (20.8%±10.2% triple therapy vs 9.2%±4.4% radiation, p=0.0308 vs 9.7%±2.9% AZD6738 plus radiation, p=0.0392; vs 9.6%±2.7% radioimmunotherapy, p=0.0369; figure 5H).

We additionally examined the percentage of TIL CD4^+^ T cells that produced IFN-γ. No differences were observed between radioimmunotherapy and triple therapy at day 8 (online supplementary figure 6A). At day 14, the percentage of TIL CD4^+^ T cells that produced IFN-γ in triple therapy was significantly higher than that in each treatment groups (51.0%±22.9% triple therapy vs 10.0%±2.2% radiation, p=0.0003; vs 16.0%±4.8% AZD6738 plus radiation, p=0.0012; vs 13.9%±0.9% radioimmunotherapy, p=0.0007; online supplementary figure 6A). Meanwhile, no differences in the percentage of splenic CD4^+^ T cells that produced IFN-γ were observed between radioimmunotherapy and triple therapy at any time points (online supplementary figure 6B). In summary, the addition of AZD6738 to radioimmunotherapy significantly increased the percentage of CD8^+^ and CD4^+^ T cells that produced IFN-γ in tumors and the spleen.

### AZD6738 promotes the antitumor effect of radioimmunotherapy via activating cGAS/STING signaling pathway

**Figure 6:**
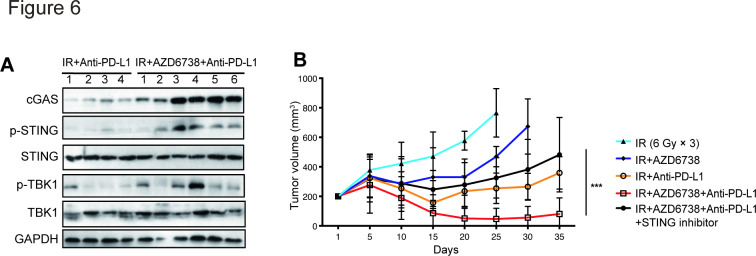
AZD6738 promotes radioimmunotherapy antitumor effect via activating cGAS/STING signaling pathway. (A) Several key proteins of cGAS/STING pathway were detected in Hepa 1–6 subcutaneous tumors from mice treated with radioimmunotherapy or triple therapy group. (B) Response of the Hepa 1–6 subcutaneous tumors to the indicated treatments. n=12 in each group. Data represent the mean±SEM. Statistical analysis was performed by using mixed-effects model, followed by Tukey’s multiple comparison test. ***p<0.0005. IR, ionizing radiation.

We then explored the mechanism underlying the synergistic effect of AZD6738 and radioimmunotherapy. Previous studies showed that radiation-induced DNA damage activates cGAS/STING signaling; therefore, we hypothesized that AZD6738, as a DDR inhibitor, might play a role in enhancing cGAS/STING activation. Analysis of levels of several key factors involved in cGAS/STING signaling in Hepa 1–6 subcutaneous tumors from mice treated with radioimmunotherapy or triple therapy revealed that AZD6738 plus radioimmunotherapy treatment increased levels of cGAS, p-STING, and p-TBK1, suggesting that AZD6738 increased activation of the cGAS/STING pathway (figure 6A). Furthermore, treatment of Hepa 1–6 tumor-bearing mice with the STING inhibitor C-176 impaired the antitumor efficacy of triple therapy (482.4±72.9 (triple therapy plus STING inhibitor) vs 80.5±31.4 (triple therapy), p<0.0001; vs 265.2±27.4 (radioimmunotherapy), p=0.0003) (figure 6B). These data indicate that AZD6738 exerted a synergistic antitumor effect with radioimmunotherapy by activating cGAS/STING signaling.

### Triple therapy enhances immune memory activation in mice tumors and spleens

**Figure 7:**
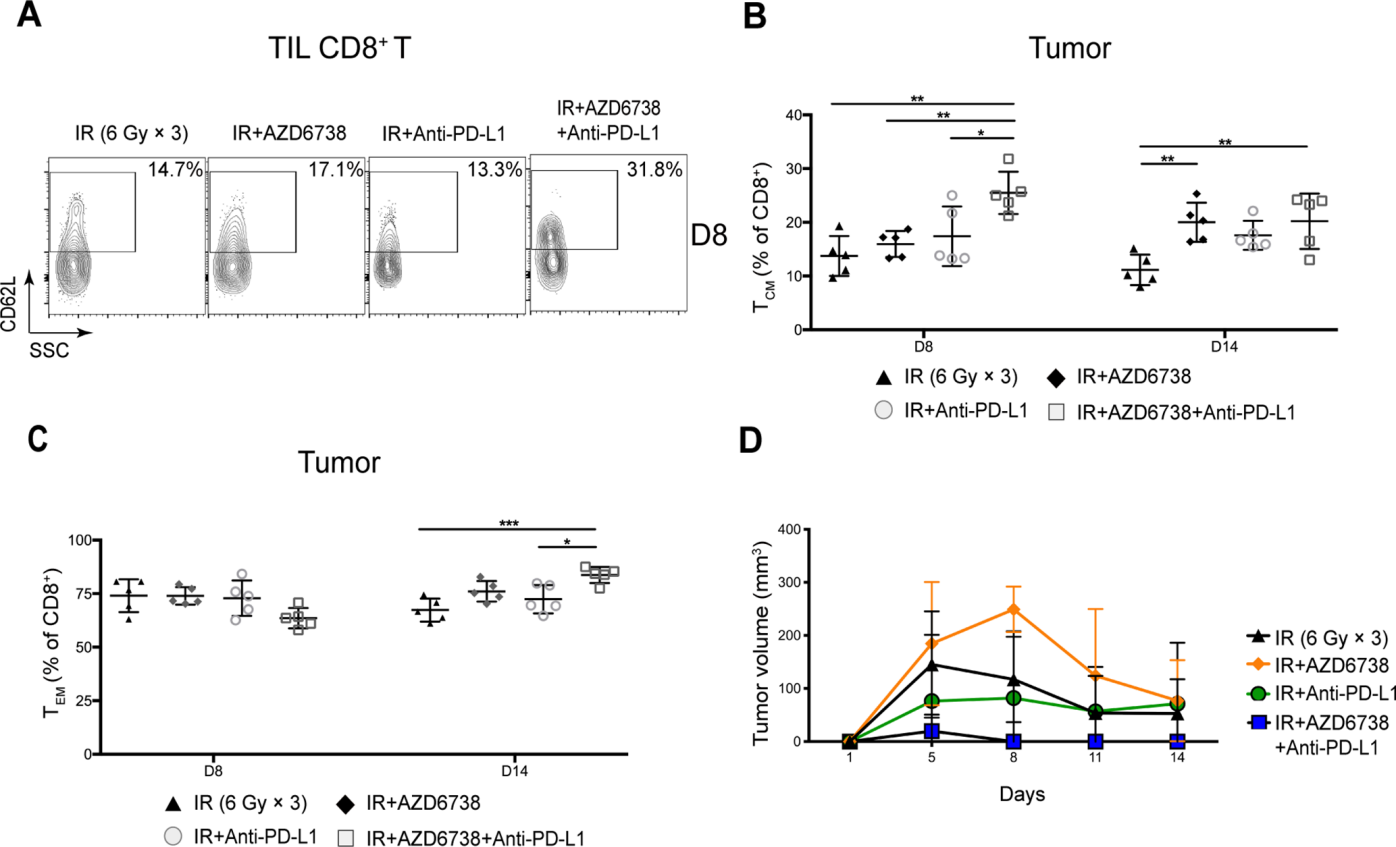
Triple therapy promotes immune memory activation in mice tumors. (A) Representative contour plots depicting CD62L and CD44 expression in tumor-infiltrating lymphocyte (TIL) CD8^+^ T cells at day 8. (B–C) Quantitation of the percentage of TIL CD8^+^ T cell central memory (T_CM_, CD62L^+^CD44^+^) cells or effector memory (T_EM_, CD62L^-^CD44^+^) cells at days 8 and 14. (D) Mice tumors were re-challenged with Hepa 1–6 cells in the contralateral flank on the 20th day. Data represent the curve for the mean tumor volumes. Data represent the mean±SD. For (B) and (C), statistical analysis was performed by using one-way analysis of variance with Tukey’s multiple comparisons test. For (D), statistical analysis was performed using mixed-effects model, followed by Tukey’s multiple comparison test. *p<0.05; **p<0.005; ***p<0.0005. IR, ionizing radiation.

We analyzed the memory status of CD8^+^ and CD4^+^ Eff T cells in mice tumors and spleens. At day 8, compared with radioimmunotherapy, triple therapy increased the percentage of TIL CD8^+^ central memory T (T_CM_) cells (25.5%±3.9% triple therapy vs 17.4%±5.6% radioimmunotherapy, p=0.0287; figure 7A, B). In addition, triple therapy reduced the percentage of TIL CD8^+^ effector memory T (T_EM_) cells (63.5%±4.8% triple therapy vs 75.4%±6.9% radioimmunotherapy, p=0.0433). At day 14, no differences in the percentage of TIL CD8^+^ T_CM_ were observed between radioimmunotherapy group and triple therapy group (figure 7B). The percentage of TIL CD8^+^ T_EM_ cells in triple therapy was significantly elevated compared with that in radioimmunotherapy at day 14 (83.7%±3.8% triple therapy vs 72.4%±6.6% radioimmunotherapy, p=0.0169; figure 7C). No significant differences in activation or memory status of TIL CD4^+^ T cells were observed between radioimmunotherapy group and triple therapy group at any time points (online supplementary figure 7).

At day 14, we found there was no differences in the percentage of splenic CD8^+^ T_CM_ cells between radioimmunotherapy group and triple therapy group (online supplementary figure 8A). Meanwhile, the percentage of splenic CD8^+^ T_EM_ cells was higher in triple therapy group compared with that in radioimmunotherapy group (43.6%±16.2% triple therapy vs 22.0%±2.7% radioimmunotherapy, p=0.0065; online supplementary figure 8B). Similar effects were seen in the percentage of splenic CD4^+^ T_CM_. The percentage of splenic CD4^+^ T_CM_ cells in triple therapy increased over time, and it was significantly higher in triple therapy than that in the other treatments at day 14 (online supplementary figure 8C). With respect to the percentage of splenic CD4^+^ T_EM_ cells, it was significantly higher in triple therapy group than that in radioimmunotherapy group at day 8 (55.7%±7.3% triple therapy vs 20.7%±2.7% radioimmunotherapy, p<0.0001; online supplementary figure 8D). Meanwhile, no differences in the percentage of splenic CD4^+^ T_EM_ were observed between radioimmunotherapy and triple therapy at day 14 (online supplementary figure 8D).

As the activation and memory status of CD8^+^ and CD4^+^ Eff T cells is closely related to tumor control, next we determined the effect of AZD6738 on tumor recurrence. We re-challenged the treatment completed tumor-bearing mice with Hepa 1–6 cells in the contralateral flank on the 20th day, and then monitored the tumor volumes of these mice treated with different regimens. We found that, compared with radioimmunotherapy, triple therapy showed a more potent inhibitory effect on the secondary tumor growth (figure 7D). These findings indicated that triple therapy enhanced immunologic memory and prevented recurrence of HCC by boosting T_CM_ and T_EM_ cells in mice tumors and spleens.

## Discussion

In our study, we found that radiation obviously upregulated the expression of PD-L1 in HCC cells, increased the number of Tregs, the CD8^+^/CD3^+^ T lymphocyte ratio and the amount of IFN-γ^+^CD8^+^ T lymphocytes in subcutaneous tumor immune microenvironment. Previous studies have shown that radiation activated CD8^+^ T cells in tumor tissues by killing tumor cells and releasing antigens from these cells.^22^ However, besides the positive effect on tumor immune microenvironment, radiation also improved to confer negative effect by activating DDR signal pathway, then upregulating PD-L1 expression in tumor cells and increasing infiltration of inhibitory lymphocytes (such as Tregs) in tumor tissues.^23–26^ These studies suggest that radiation has dual roles in modulating tumor immune microenvironment, which is consistent with our findings.

The regulative role of radiation in tumor immune microenvironment provides the rational for radioimmunotherapy, which has been widely studied in the field of tumor therapy.^27^ Numerous preclinical studies have shown the promising antitumor effect of radioimmunotherapy in several cancers including lung cancer, melanoma, and pancreatic cancer, by improving the tumor immune microenvironment.^27–30^ Moreover, two preclinical studies have shown that radioimmunotherapy increased the number and IFN-γ secretion of TIL CD8^+^ T cells and improved the local control rate of HCC.^31^ Importantly, several clinical trials regarding lung cancer have indicated the benefit of radioimmunotherapy on patient’s survival. For example, the PACIFIC trial and KEYNOTE-001 trial revealed that ICIs improved progression-free survival and overall survival of patients with unresectable stage III non-small cell lung cancer (NSCLC) who previously received chemoradiotherapy.^32 33^ Even though radioimmunotherapy displayed a promising antitumor treatment benefit on NSCLC, clinical trials to investigate the efficacy of radioimmunotherapy on patients with HCC are still ongoing and the results are immature.

Radiation-induced DNA damage promotes the expression of immunosuppressive molecules including PD-L1 and increases immunosuppressive cells in tumor tissues, by activating ATR signaling pathway.^15^ It is well documented that, compared with radiation alone, the addition of AZD6738 increased the number and the IFN-γ secreting ability of TIL CD8^+^ T lymphocytes.^16 17^ However, as for the Hepa 1–6 subcutaneous xenograft model in our study, we found that although there is a trend that tumor control rate and survival of mice treated with radiation plus AZD6738 was higher than the mice treated with radiation alone, the difference did not reach statistical significance. These findings are inconsistent with what Vendetti *et al* reported that radiotherapy combined with AZD6738 prolonged the survival time of CT26 tumor-bearing mice.^16^ Although such discrepancy remains unclear, one possible explanation for it may be due to the distinct cancer types we focused. In our case, we studied HCC, whereas Vendetti *et al* studied colorectal cancers. However, as for the alterations of TILs, we found that, compared with radiation alone, AZD6738 plus radiation significantly decreased the number of Tregs, increased the ratio of CD8^+^/CD3^+^ T cells, and the number of IFN-γ^+^CD8^+^ lymphocytes in Hepa 1–6 tumor tissues. Although the survival time of mice was not extended by the addition of AZD6738 to radiation, tumor immune microenvironment was significantly improved, suggesting that AZD6738 could be a suitable synergistic treatment for radioimmunotherapy. In our study, we found that the combination of AZD6738 with radioimmunotherapy was not only well tolerated by HCC tumor-bearing mice, but also conferred better tumor control and prolonged mice survival.

It has been well reported that radiation combined with ICIs had a strong immunostimulatory effect, including increasing the number of CD8^+^ T cells, enhancing the activity of CD8^+^ T cells, and reducing the number of immunosuppressive cells such as Tregs. Consistent with these findings, the similar phenomenon was shown in our case, which may contribute to the better tumor control and survival in mice treated with radiation combine with anti-PD-L1 compared with the mice treated with radiation alone. Intriguingly, in our study, we found that the levels of CD8^+^ T cells and IFN-γ^+^CD8^+^ T cells remained the same at days 8 and 14. However, the addition of AZD6738 to radioimmunotherapy progressively boosted the infiltration and activation of CD8^+^ T cells with time in mice tumor tissues. These findings implied that AZD6738 has the potential ability to mediate robust immunostimulatory effects that could synergize with radioimmunotherapy in tumor control and survival improve.

AZD6738 was reported to trigger innate immune cell infiltration through activation of cGAS/STING pathway, which shed light on the possibility that the blockage of cGAS/STING signaling pathway could weaken the antitumor effect of AZD6738.^17^ In our study, we found that the combination of AZD6738 with radioimmunotherapy led to the activation of cGAS/STING pathway. More importantly, STING inhibitor C-176 impaired the antitumor efficacy of triple therapy in tumor-bearing mice.

T_CM_ cells play a very important role in preventing tumor recurrence and metastasis. Due to the self-renew property and the long survival in vivo, T_CM_ cells are able to be rapidly activated and differentiate into effective T cells on the re-exposure stimulation of tumor antigens. As a result, tumor cells are killed, and tumor recurrence or metastasis is prevented.^34 35^ Thus, the increase in number of memory T cells can provide better protection for patients with cancer. Several studies have shown that radiation combined with ICIs or AZD6738 increased the number of T_CM_ cells and prevent tumor recurrence and metastasis.^16 27^ In our study, compared with radioimmunotherapy, the percentage of TIL CD8^+^ T_CM_ cells was higher following triple therapy, which may contribute to the delayed tumor recurrence in mice treated with triple therapy.

Several clinical studies are being conducted to explore the effect of ATR inhibitors combined with DNA-damaging therapeutic interventions including radiotherapy or chemotherapy in varied types of tumors.^36–38^ In this study, ATR inhibitor AZD6738 did not enhance the HCC control rate of radiotherapy. However, AZD6738 dramatically promoted radioimmunotherapy-induced CD8^+^ lymphocyte infiltration and activity, reduced the number of Tregs, and increased memory T cell infiltration in HCC xenograft tumors. Remarkably, AZD6738 in combination with radioimmunotherapy was well tolerated, prolong survival, and delayed recurrence of the tumor-bearing mice.

Taken all these benefits of AZD6738 combined with radioimmunotherapy together, our data implicated that AZD6738 could be a promising synergetic treatment for radioimmunotherapy in patients with HCC. Although our study was of high clinical significance, the combination of AZD6738 with radioimmunotherapy merits evaluation on clinical trials in patients with HCC.

## Conclusions

In summary, we have demonstrated that the addition of AZD6738 to radioimmunotherapy increased the infiltration of CD8^+^ T cells, IFN-γ^+^CD8^+^ and CD4^+^ T cells, T_CM_ cells, and T_EM_ cells in Hepa 1–6 tumors. Moreover, the combination of AZD6738 with radioimmunotherapy led to a descending trend in the number of TIL Tregs and exhausted T cells. Thus, AZD6738 significantly improved the tumor immune microenvironment following radioimmunotherapy. In addition, AZD6738 caused the similar immunophenotype in mice spleens. As a consequence, compared with radioimmunotherapy, AZD6738 plus radioimmunotherapy displayed better therapeutic efficacy in inhibiting tumor growth, prolonging survival, and preventing recurrence in tumor-bearing mice.

Our findings have a high clinical significance. Based on preliminary translational observations, our work raised the possibility that AZD6738 might be a potential synergistic modality for radioimmunotherapy in HCC. Remarkedly, this study provides a basis for future clinical human studies into the treatment of HCC.
