# Supplementary material for: ATR inhibitor AZD6738 enhances the antitumor activity of radiotherapy and immune checkpoint inhibitors by potentiating the tumor immune microenvironment in hepatocellular carcinoma
Source: J Immunother Cancer. 2020 May 26;8(1):e000340. doi: 10.1136/jitc-2019-000340 (PMC7254123; doi:10.1136/jitc-2019-000340)
Supplement: Supplementary data [file jitc-2019-000340supp001.pdf]

Supplementary Methods

Lymphocytes extraction

Spleens and tumors of the mice were harvested in RPMI medium with 10% FBS. The tumor tissue was chopped into small pieces, harvested in RPMI medium supplemented with 0.5mg/ml collagenase IV (Solarbio Life Science) and 0.1mg/ml deoxyribonuclease type I (Solarbio Life Science), incubated at 37°C for 1 hour, then mechanical dissociated on a frosted glass slide. The harvested spleen was directly mechanically dissociated on a frosted glass slide. The tumor or spleen cell suspension was filtered through a 70-μm cell strainer (Corning) to obtain a single-cell suspension. Red blood cells (RBCs) were lysed with 1 mL RBC lysis buffer (150 mM NH_4Cl, 10 mM $NaHCO_3$, 0.1 mM EDTA) for 1 minute at room temperature, after which the lysate was quenched using 5 mL RPMI. The cell suspension was counted using Cellometer Auto T4 (Nexcelom) or Scepter (Millipore), and 1×10^6 cells were seeded in a 6-well round bottom plates for subsequent experiments. For cytokine analysis, Leuko Act Cktl With GolgiPlug (eBioscience Inc.) was added to the cell culture medium to stimulate the lymphocytes for 4-6 hours, and the cells were further cultured for 3 hours for subsequent staining.

Flow cytometry analysis

The cells were stained at 4°C for 30 minutes in Buffer II (1x PBS +

1 g/ml BSA + 0.5 g/ml NaN_3) with the antibodies against certain surface antigens, fixed and permeabilized at 4°C for 50 minutes in Transcription Factor Buffer Set (eBioscience), and then stained for intracellular protein flow cytometry at 4°C for 50 minutes in 1x perm/wash buffer (BD Biosciences). Prior to surface staining and fixation, cells were washed with Buffer II and 1x perm/wash buffer in Transcription Factor Buffer Set (eBioscience) before and after intracellular staining. Uncompensated data were collected using a LSRFortessa cell counter with FACSDiva software (BD Biosciences). Compensation and analysis were performed using FlowJo v10.4.0 software. Splenocytes were used for all other compensation controls. The following antibodies (clones, catalogue number) were purchased from BioLegend: FITC-CD3 (17A2, 100204), Brilliant Violet 510™-CD8a (53-6.7, 100752), PE-CD25 (PC61, 102008), Brilliant Violet 421™-FOXP3 (MF-14, 126419), APC-IFN-γ (XMG1.2, 505810), PE/Dazzle™-594 Ki-67 (16A8, 652428), APC-CD62L (MEL-14, 104412), PE/Dazzle™-594 CD44(IM7, 103056), APC/Cy7-CD279 (PD-1)(29F.1A12, 135224), PE-CD366 (Tim-3) (B8.2C12, 134004), Brilliant Violet 421™-CD223 (LAG-3) (C9B7W, 125221), PerCP/Cy5.5-CD274 (B7-H1, 124334), PD-L1 (10F.9G2, 124334). Gating strategies are shown in Supplementary Figure 1.

Analysis of Hepal-6 PD-L1 expression *in vitro*

Hepal-6 cells were treated with AZD6738 (300 nM) or DMSO vehicle (0.25%) immediately prior to receiving 2Gy radiation. The cells were stained with anti-PD-L1 antibody PerCP/Cy5.5 (10F.9G2 , BioLegend) at 4°C for 30 minutes. Analysis was performed using a LSRFortessa cell counter with FACSDiva software (Biosciences). After gating with SSC-A and FSC-A, the PD-L1 mean fluorescence intensity of the cells was determined.

Supplementary Figures

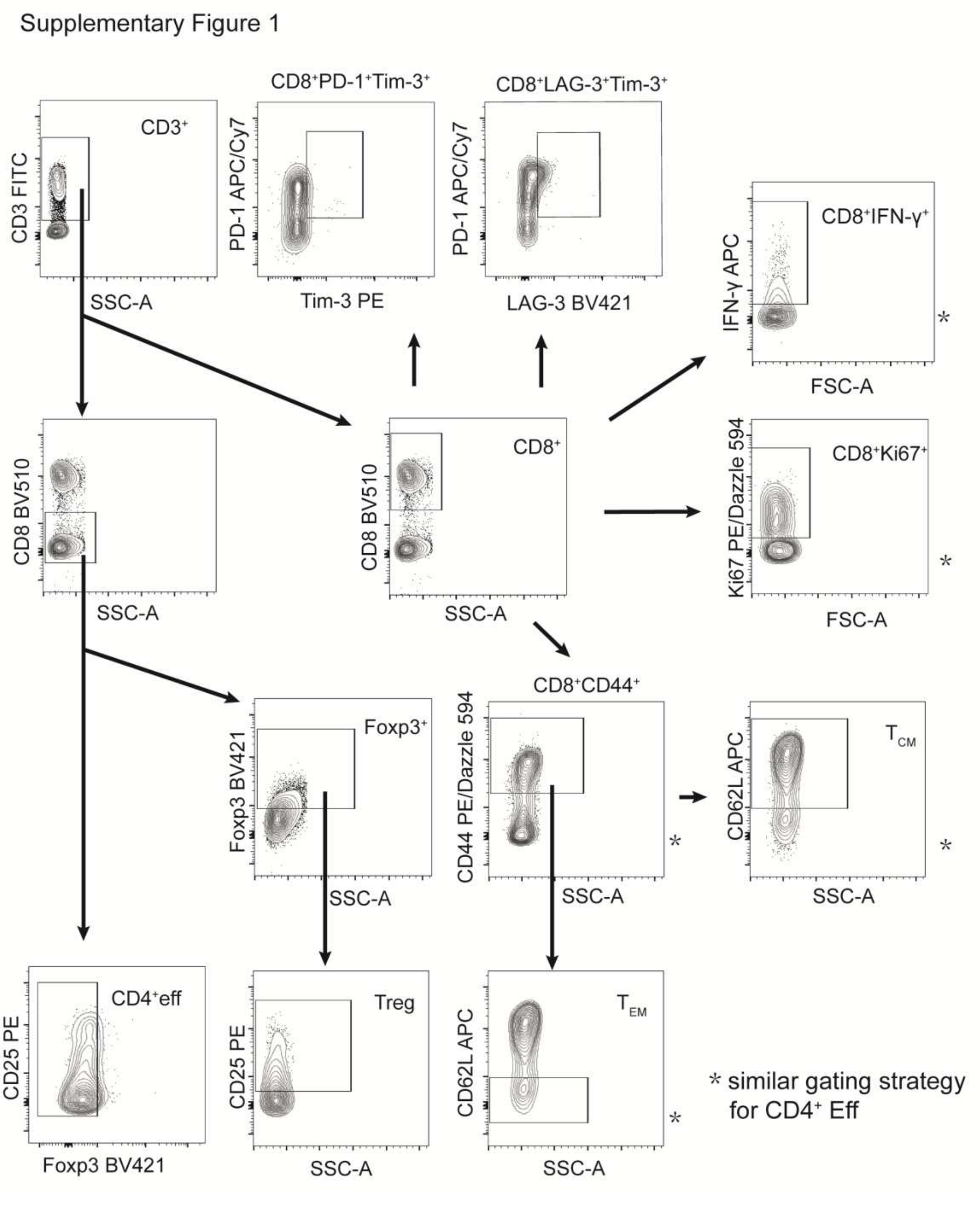

Supplementary Figure 1. Gating strategy for analysis of spleen- and tumor-infiltrating T cells in Hepa1-6 tumor-bearing mice. Exclusion of unstable portions of the run and doublets was completed before the gating. Cells were gated based on the characteristics of SSC-A and FSC-A. CD3^{+}

cells were characterized as $CD8^+$ T cells ($CD8^+$) or $CD4^+$ T cells ($CD8^-$). $CD4^+$ cells were characterized as $CD4^+$ Eff T cells ($Foxp3^-$) or Tregs ($CD25^+$ $Foxp3^+$). For $CD8^+$ and $CD4^+$ Eff T cells, expression of IFN-γ and Ki67 was examined, and cells were characterized as naïve (TN, $CD62L^+CD44^-$), central memory (T_{CM}, $CD62L^+CD44^+$) and effector memory (T_{EM}, $CD62L^-CD44^+$) populations. Expression of PD-1, LAG-3, and Tim3 was also examined on $CD8^+$ T cells. Asterisk indicates the similar gating strategy for $CD4^+$ Eff T cells. Gates were established using spleens of vehicle control mice.

Supplementary Figure 2

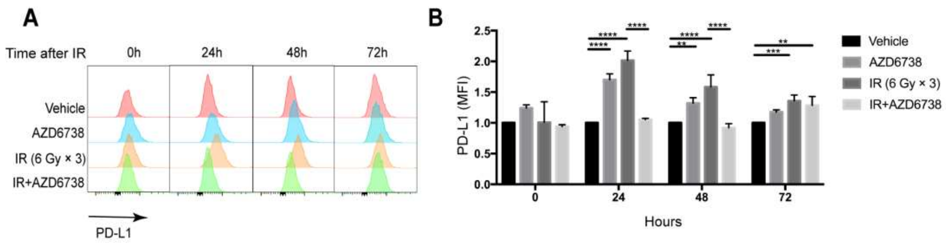

Supplementary Figure 2. Inhibition of the DDR pathway inhibits upregulation of PD-L1 expression in Hepal-6 cells after radiotherapy. (A-B) Representative histograms of PD-L1 expression in Hepal-6 tumor cells (A) and quantitation of PD-L1 median fluorescence intensity (B). Data represent the mean ± SD. For (B), statistical analysis was performed by using one-way ANOVA with Tukey's multiple comparisons test. **p<0.005, ***p<0.0005, ****p<0.0001.

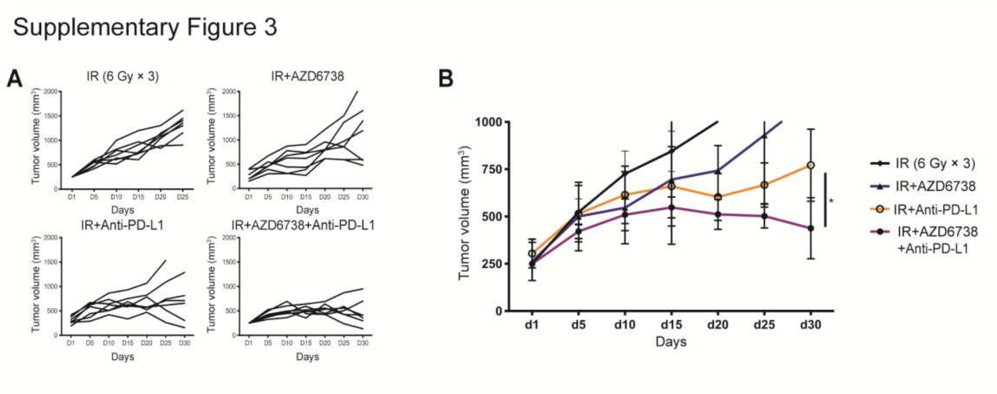

Supplementary Figure 3. Addition of AZD6738 improve the tumor growth control of radioimmunotherapy in H22 tumor-bearing mice. (A-B) Response of individual tumor (A) or the subcutaneous tumors (B) to the indicated treatment regimens. n=7 in each group. Data represent the mean ± SEM. For (B), statistical analysis was performed by using mixed-effects model, followed by Tukey's multiple comparison test. *$p < 0.05$.

Supplementary Figure 4

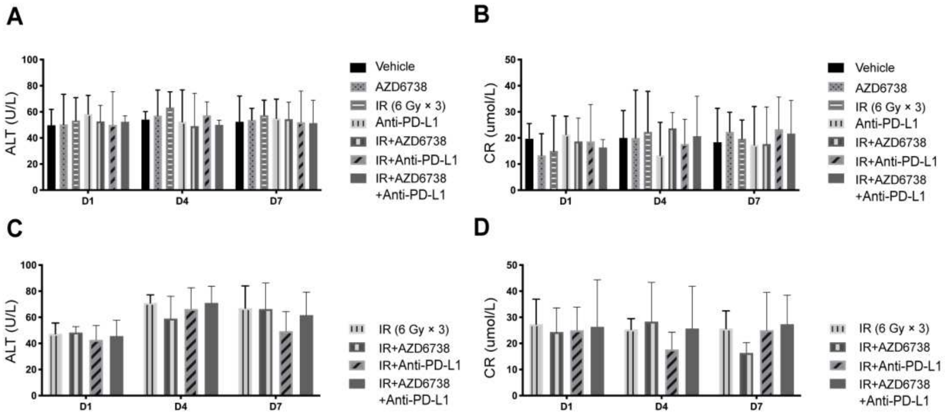

Supplementary Figure 4. Triple therapy did not cause liver and kidney dysfunctions in mice. (A-B) Peripheral blood ALT (A) and Scr (B) from Subcutaneous Hepa1-6 tumor-bearing mice on the 1st, 4th and 7th days after the start of treatment. (C-D) Peripheral blood ALT (C) and Scr (D) from orthotopic Hepa1-6 bearing-mice on the 1st, 4th and 7th days after the start of treatment. Data represent the mean ± SD. Statistical analysis was performed by using one-way ANOVA with Tukey’s multiple comparisons test.

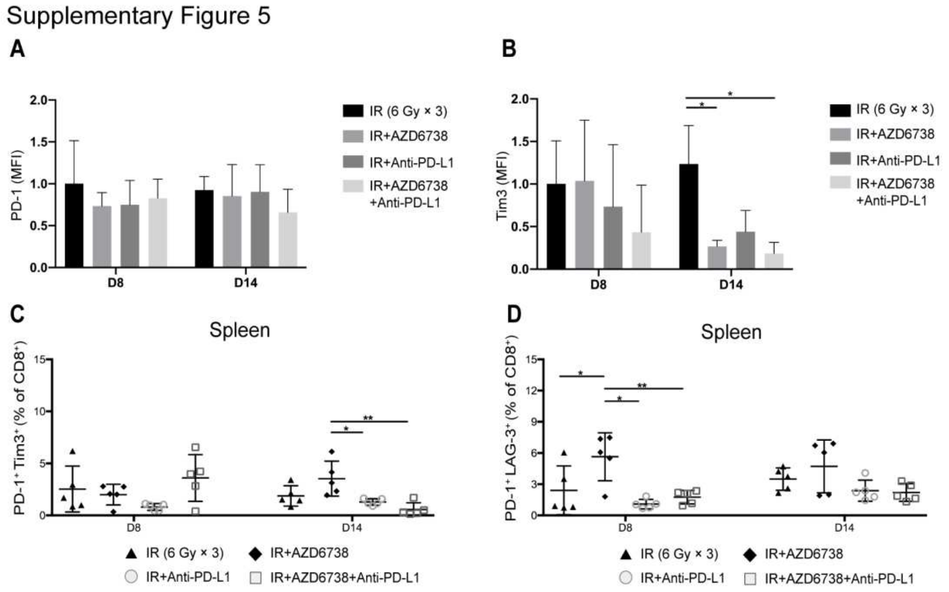

Supplementary Figure 5. Triple therapy attenuates co-expression of splenic CD8^{+} T cell exhaustion markers. (A-B) Quantitation of PD-1 (A) and Tim3 (B) median fluorescence intensity in TIL CD8^{+} T cells at days 8 and 14. (C-D) Quantitation of the percentage of splenic CD8^{+} T cells that co-express PD-1 and Tim-3 (C) and co-express PD-1 and LAG-3 (D) on days 8 and 14. Data represent the mean ± SD. Statistical analysis was performed by using one-way ANOVA with Tukey's multiple comparisons test. *p<0.05, **p<0.005.

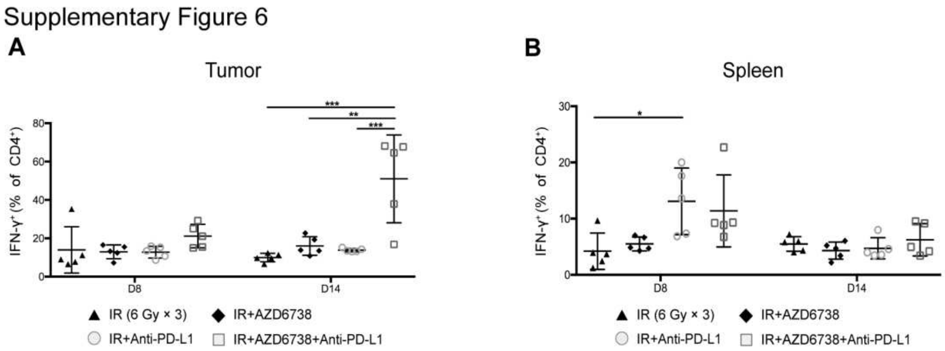

Supplementary Figure 6. Triple therapy promotes $CD4^+$ T cell effector function. Quantitation of the percentage of TIL (A)and splenic (B) $CD4^+$ T cells that express IFN-γ on days 8 and 14. Data represent the mean ± SD. Statistical analysis was performed by using one-way ANOVA with Tukey's multiple comparisons test. *p<0.05, **p<0.005, ***p<0.0005.

Supplementary Figure 7

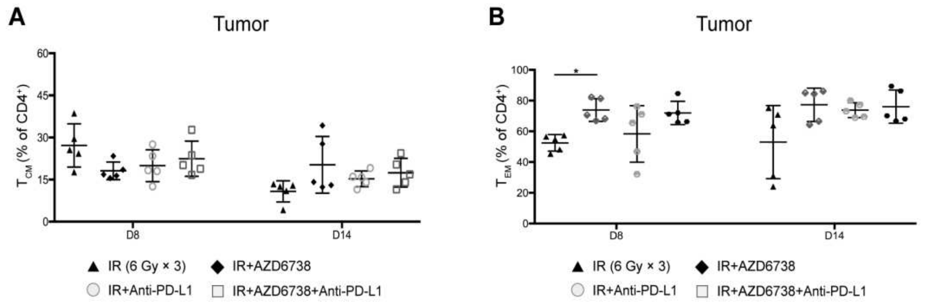

Supplementary Figure 7. Triple therapy promotes tumor immune memory activation. Quantitation of the percentage of TIL $CD4^+$ T cells, central memory (T_{CM}, $CD62L^+CD44^+$) (A), or effector memory (T_{EM}, $CD62L^-CD44^+$) (B) phenotypes on days 8 and 14. The mean and SD bars are shown. Statistical analysis was performed by using one-way ANOVA with Tukey's multiple comparisons test. *p<0.05.

Supplementary Figure 8

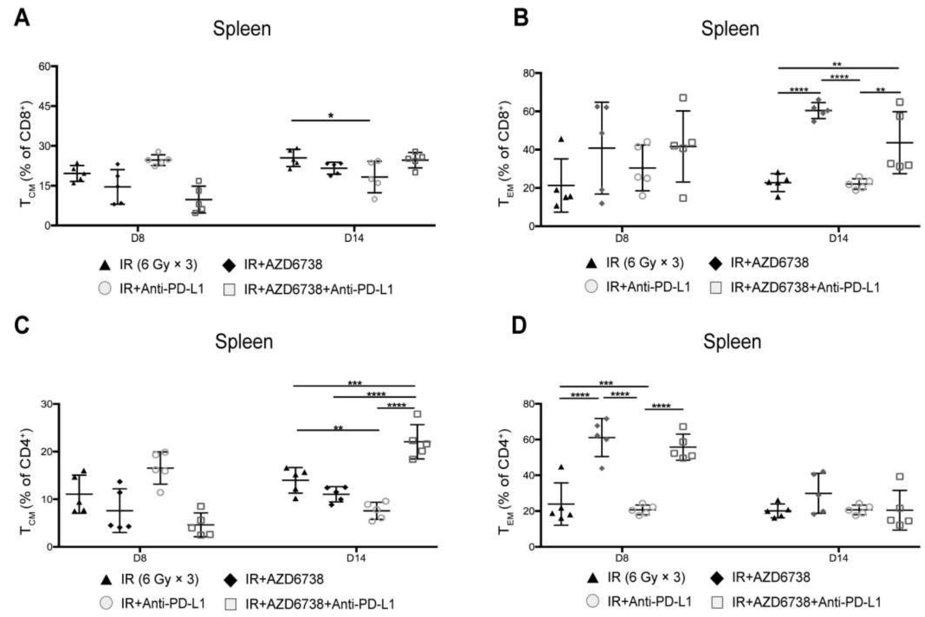

Supplementary Figure 8. Triple therapy promotes splenic immune memory activation. (A-B) Quantitation of the percentage of splenic $CD8^+$ T_{CM} (A) and T_{EM} (B) cell phenotypes on days 8 and 14. (C-D) Quantitation of the percentage of splenic $CD4^+$ T_{CM} (C) and T_{EM} (D) cell phenotypes on days 8 and 14. The mean and SD bars are shown. Statistical analysis was performed by using one-way ANOVA with Tukey's multiple comparisons test. *p<0.05, **p<0.005, ***p<0.0005, ****p<0.0001.
